# Quantification of coronary enhancement - reproducibility of methods and feasibility of quantification in health and disease

**DOI:** 10.1186/1532-429X-15-S1-P84

**Published:** 2013-01-30

**Authors:** Niharika Varma, Rene M Botnar, Andreas Indermuehle, Sarah A Peel, Gerald F Greil, Eike Nagel, Valentina O Puntmann

**Affiliations:** 1 Cardiovascular Imaging, King's College London, London, UK; 2 Department of Medical Physics and Bioengineering, Division of Imaging Sciences and Biomedical Engineering, King's College London, London, UK

## Background

Coronary enhancement (CE) imaging by magnetic resonance (MR) is a novel, non-invasive approach for visualization of contrast uptake within the coronary artery vessel wall. Quantification of CE may help to individualize sub-populations at risk for the benefit of early risk assessment and intervention. Here we sought to compare the reproducibility of several quantification methods and to investigate the feasibility to detect differences in healthy subjects and disease.

## Methods

**Figure 1:**
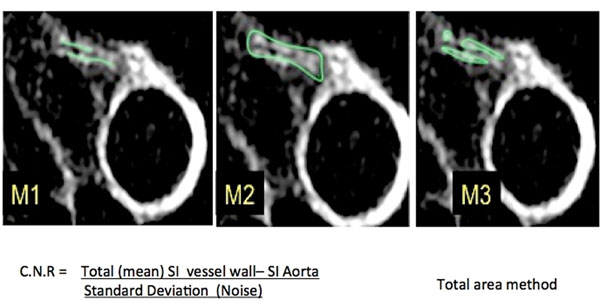


All imaging was performed with a 3T MRI scanner. Targeted volume coronary imaging was performed using double-oblique imaging planes parallel to either the left and right coronary artery defined by a 3-point plan-scan tool. A balanced steady state free precession sequence (acquired in-plane resolution=1.25x1.25x3mm; TR/TE/FA: 4.2ms/1.5ms/110°) was used for visualization of the lumen. Subsequently, inversion-recovery T1 weighted 3D gradient echo coronary imaging (TR/TE/FA: 6.1msec/1.9 msec/30°) was performed 40 minutes after administration of 0.2 mmol/kg of gadobutrol. Three methods of quantification M1-M3, were applied to the proximal coronary artery of each subject's dataset. M1 and M2 generated a mean contrast to noise ratio (CNR) by using coronary and aortic blood signal intensity (SI): M1 only included the visually detectable enhancement in the wall of each arterial segment whereas M2 included the complete segment (lumen and wall) and derived an average SI per segment (Figure 1). M3 was used to quantify a 'total visually detectable area' of CE. Analysis was performed by two independent observers for inter and intra-observer reproducibility. We then tested the feasibility of these methods to generate values in healthy subjects and those with either coronary or systemic inflammatory disease.

## Results

Analysis could be performed in 100 and 93% of thirty subjects for M1-2 and M3, respectively. M1 and M3 both showed reasonable overall intraobserver agreement (M1: r=0.91; P<0.01, mean difference (MD)±SD=-0.04±1.1; M3: r=0.89, P<0.01, MD±SD=-0.2±0.4). Between observers, M1 and M3 showed good reproducibility (M1: r=0.88, P<0.01; M3: MD±SD=0.5±2.8 r=0.75, MD±SD=2.1±1.4, P<0.01). Method M2 showed inferior intra and inter-observer agreement. Using M1 and M3, there was a significant difference between healthy subjects (n=7), CAD (n=10) and SLE (n=13) patients, respectively (M1, CNR (mean±SD) 1.9±0.8 vs. 5.4±1.9 vs. 6.4±1.3, one-way ANOVA: P<0.01; M3, mm2: 1.03±0.9 vs. 3.8±2.2 vs. 3.2±1.4, P=0.002. Using M2, no distinction between healthy subjects and patients could be made (p=0.2).

## Conclusions

Quantification of visualized CE in proximal coronary segments using CNR and total area is feasible and reproducible. Both methods are able to discern significant differences between health and disease.

## Funding

NIHR Biomedical Research Centre award (Atherosclerosis theme).

